# Too deductive too soon? Toward an inductive renewal of implementation science

**DOI:** 10.1186/s13012-026-01504-4

**Published:** 2026-05-04

**Authors:** Per Nilsen, Roman Kislov, Sarah A. Birken, Brian S. Mittman

**Affiliations:** 1 Department of Health, Medicine and Caring Sciences, Linköping University, Linköping, Sweden; 2 Faculty of Business and Law, Manchester Metropolitan University, Manchester, UK; 3 School of Health Sciences, The University of Manchester, Manchester, UK; 4 Department of Implementation Science, Wake Forest University School of Medicine, Winston-Salem, NC USA; 5 Department of Research & Evaluation, Kaiser Permanente Southern California, Pasadena, CA USA

**Keywords:** Implementation science, Theory development, Inductive methods, Abductive reasoning, Qualitative inquiry, Theories, models, and frameworks, Conceptual innovation, Methodological pluralism

## Abstract

**Background:**

Over the past two decades, implementation science has developed a strong conceptual foundation through the proliferation and widespread use of theories, models, and frameworks (TMFs). These have provided coherence, shared vocabulary, and methodological discipline across a rapidly expanding field. However, this success has also produced an unintended consequence: increasing reliance on deductive modes of inquiry, in which a limited set of established TMFs are repeatedly applied as analytic templates across diverse empirical contexts. This tendency toward early deductivism risks constraining theoretical development, reducing sensitivity to heterogeneity, complexity, and temporality inherent in implementation, and reinforcing methodological circularity.

**Toward an inductive renewal of implementation science:**

In this conceptual paper, we argue for an inductive renewal of implementation science that rebalances deduction with stronger inductive and abductive forms of reasoning. Rather than abandoning established TMFs, we propose reframing them as evolving heuristics – resources for structuring inquiry that remain open to refinement, extension, and selective reconfiguration through empirical engagement. We clarify the complementary roles of induction, abduction, and deduction in theory development, emphasizing abductive iteration as a mechanism for translating empirical discovery into cumulative conceptual advancement. We outline strategies for advancing this agenda across three interdependent levels. At the study level, this involves treating TMFs as provisional heuristics, re-embracing qualitative discovery, and using abductive reasoning to refine theory through engagement with unexpected findings. At the field level, shared infrastructures for synthesis and longitudinal learning are needed to support cumulative, context-sensitive theorizing and to account for the temporal dynamics of implementation. Institutionally, journals and funders must recalibrate incentives to value theory development, adaptation, and transparency alongside theory application. Drawing on examples from research on knowledge brokering and implementation scale-up, we show how theoretically informative contributions emerge when empirical surprises, temporal dynamics, and analytic tensions are used to interrogate and refine existing TMFs rather than being absorbed into pre-specified categories.

**Conclusion:**

A mature implementation science must move beyond asking which TMF best fits a study, toward examining how empirical phenomena challenge, extend, and reshape theory. Sustaining this balance is essential for theoretical coherence and continued conceptual innovation.

**Table:** 

**Contributions to the literature**
• Implementation science’s reliance on deductive application of a small set of theories, models, and frameworks (TMFs) limits sensitivity to contemporary implementation challenges. • We propose a renewed emphasis on inductive and abductive reasoning, showing how these logics can complement existing deductive approaches to generate richer, more context-sensitive theory. • A re-inductive agenda for implementation science is presented by aligning study-level methodological change, field-level infrastructures for cumulative theory-building, and institutional reforms that enable adaptive, practice-grounded theorizing. • Rethinking TMFs as tools for ongoing refinement, rather than templates for repeated application, offers a pathway for preserving theoretical coherence and revitalizing the capacity for conceptual development.

## Background

Implementation science in health emerged as a consolidated field in the early 2000s in response to a persistent problem: the slow and uneven integration of evidence-based innovations into real-world practice [1]. To address this gap between research and practice, scholars expanded the previously limited set of theories, models, and frameworks (TMFs), including the Ottawa Model of Research Use [2] and the Promoting Action on Research Implementation in Health Services framework (PARIHS) [3].

Building on this foundational expansion, the field was further shaped by a series of influential complementary contributions, such as the implementation outcomes taxonomy [4], the Consolidated Framework for Implementation Research (CFIR) [5], Normalization Process Theory (NPT) [6], the Theoretical Domains Framework (TDF) [7], the Expert Recommendations for Implementing Change (ERIC) [8], and the Nonadoption, Abandonment, Scale-up, Spread and Sustainability framework (NASSS) [9]. These TMFs strengthened and legitimized the emerging discipline by helping researchers name key constructs, articulate implementation determinants, strategies, and outcomes, and organize an increasingly complex body of findings [10].

Yet, two decades later, the field seems increasingly constrained by earlier conceptual commitments. The same TMFs that established the field’s conceptual boundaries may now be constraining its evolution. We argue that implementation science has become excessively deductive, favoring the repetitive application of early TMFs over open-ended, inductive, or abductive exploration necessary for translating the interplay between the empirical and the theoretical into genuinely novel contributions [11]. The result is a discipline that risks reifying its own formative theoretical approaches rather than revising them in light of new empirical knowledge.

### Deductive foundations and their emerging limits

There are good reasons for implementation science’s gravitation toward deduction. In its formative years, the field sought cumulative knowledge, a counterpoint to methodologically scattered and theoretically light traditions that limited the coherence and value of individual studies. Deductive TMFs provided a shared vocabulary that enabled researchers to compare studies and develop cumulative knowledge regarding determinants, strategies, and outcomes [8, 12, 13]. At that stage, the field needed coherence more than novelty. TMFs offered structure, methodological stability, and analytical discipline across diverse study contexts [14, 15]. Integrative classification efforts, including Nilsen’s taxonomy of TMFs [16], further supported this consolidation by clarifying conceptual distinctions and relationships within an expanding theoretical landscape.

In applied domains such as health services research, such conceptual convergence is often advantageous. A unified set of constructs, such as *fidelity*, *readiness to change*, and *leadership*, facilitates rigorous data collection and analysis, synthesis of evidence, and cumulative knowledge. Deductive reasoning also allows incremental refinement; by testing and adapting established TMFs, the field can pursue greater theoretical precision. Yet coherence comes at a cost. When achieved too early or too rigidly, deductive consensus may rest on limited empirical foundations and risk becoming an intellectual cul-de-sac [17].

Implementation scientists, like scholars in other social science disciplines, are motivated to innovate conceptually and to introduce new ideas, approaches, and TMFs. This impulse is not merely academic; it is reinforced by the persistent recognition that implementation science has not yet fully achieved its goals. Despite substantial progress, there remains broad agreement that major implementation barriers endure, evidence–practice gaps persist, and no single theoretical approach has yet provided the answer to the complexity of implementation challenges [18, 19]. As a result, continued research, creativity, and conceptual development remain both necessary and incentivized. It is therefore unsurprising that the field has produced a large and increasing number of TMFs, reflecting ongoing efforts to better understand and address these unresolved problems [20].

Despite their rapid growth, TMFs have also become a source of concern. The large number of newly introduced TMFs, combined with doubts about whether many provide meaningful additional value, has led the field to increasingly adopt a stance opposing the creation of further TMFs [13, 20]. This position reflects the assessment that many proposed TMFs are not genuinely novel, but rather restatements, recombinations, or partial subsets of existing frameworks, often narrower, less theoretically grounded, or less empirically robust than those already available. From this perspective, calls to resist further TMF development are grounded in a desire to protect coherence, avoid duplication, and encourage more disciplined use of well-established TMFs rather than continual reinvention.

There is, therefore, a legitimate and important basis for skepticism toward unchecked proliferation of TMFs. The concern is not merely numerical, but conceptual; too many frameworks that fail to offer new explanatory leverage can dilute cumulative knowledge rather than advance it. Yet stopping theoretical development altogether, or relying exclusively on existing TMFs as if they were complete or final, is not a viable solution. The key issue is not whether the field should continue to work on TMFs, but how it should do so. The central tension lies in balancing the need to keep refining, extending, and sometimes developing TMFs in response to evolving empirical realities, against the recognition that many new frameworks have added little and that conceptual innovation must meet a higher bar.

It is against this backdrop that implementation science’s current situation should be understood. The field now occupies a phase common to maturing disciplines, in which early conceptual consolidation encounters the limits of its own success [21]. As empirical richness has expanded across sectors, contexts, and sociotechnical conditions, limitations in prevailing TMFs have become increasingly visible. This increasing recognition is not an argument for indiscriminate novelty, but rather for a more reflexive, empirically grounded approach to theory development, one that acknowledges the value of existing TMFs while recognizing that continued refinement, adaptation, and, where warranted, the development of new or substantially revised frameworks remains necessary.

### The risks of early deductivism

The foundational implementation science TMFs emerged from relatively circumscribed empirical foundations, primarily qualitative studies conducted in Western health systems during the 1990s and early 2000s. Yet these early TMFs soon acquired canonical status. Their constructs now permeate research design, training curricula, and even funding templates. New researchers, urged to “anchor their studies in theory”, frequently choose from a repertoire of core TMFs, implicitly assuming that they capture the essential mechanisms governing implementation [22].

Such theoretical conservatism may have prematurely constrained the field’s explanatory horizon; TMFs rooted in early institutional experiences may be ill-suited to capture the complexity, heterogeneity, and temporality of contemporary implementation processes or the demands of 21st-century contexts. These include hybrid care models that blend in-person and digital or remote modalities; digital health ecosystems embedded within interdependent sociotechnical systems; the emergence of artificial intelligence systems requiring ongoing monitoring, data governance, and complex human–technology interaction; and academia–industry partnerships that span organizations, professions, sectors, and regions globally. When these evolving realities are forced into pre-existing categories, empirical novelty risks being lost.

Implementation science studies often begin with a chosen TMF rather than open-ended inquiry, guided by strong recommendations to ground empirical research in relevant theory. The TMF functions as a coding template, guiding observation and interpretation. Although this practice facilitates synthesis and contributes to efficiency, it discourages discovery-oriented research. By privileging verification over exploration, the field sidelines methods known for generating conceptual insights, e.g., ethnography, grounded theory, interpretive description, or systems mapping [23–25].

The consequence is methodological circularity; studies designed under a specific TMF unsurprisingly confirm the relevance of that TMF. Few challenge its boundaries or propose alternatives. Over time, a discipline built to study implementation and generate new insights risks becoming one that merely validates its own TMFs.

Prevailing TMFs aimed for broad applicability across settings, focusing less on clarifying underlying mechanisms and more on identifying general determinants. However, when these determinant-focused TMFs are reapplied deductively, their lack of explicit mechanisms can turn their intended flexibility into a liability. For example, *readiness for change*, *professional culture*, or *leadership engagement* can mean radically different things across cultural and organizational settings [26].

Beyond contextual variation, linguistic translation introduces additional risks. Translation into different languages may improve accessibility and dissemination, but it does not automatically ensure conceptual equivalence or sensitivity to local meanings. Without sustained interpretive engagement, translated constructs may still function as standardized categories rather than contextually grounded explanations.

Separately, even within a single cultural setting, constructs risk being operationalized as checklist-style variables, which can shift attention away from underlying mechanisms and lived processes toward categorical classification. This tendency to categorize and classify rather than interpret reproduces the very epistemic gap that implementation science aimed to bridge: the gap between academic knowledge and real-world practice [19].

### The case for an inductive turn: rationale and reservations

A reductive turn to reopen conceptual inquiry from empirical grounds is not a call for “anything-goes” empiricism. Rather, it recognizes that theories mature through cyclical engagement between deduction and induction [27]. As the empirical base of implementation science has expanded, the range and content of TMFs must evolve accordingly.

The inductive turn we advocate encompasses both inductive and abductive forms of inquiry. Induction refers to analytic approaches that generate concepts and explanations from empirical observations; abduction involves iterative movement between empirical findings and existing theory, using unexpected or anomalous observations to refine, extend, or reconfigure theoretical constructs [28]. Induction is essential for opening up new conceptual space, and abduction plays a crucial role in connecting emergent insights to existing theoretical traditions, thereby supporting cumulative theory development rather than fragmentation [27, 29]. Both inductive and abductive logic of inquiry are essential to enable the field of implementation science to move from merely theory-informed to theoretically informative empirical research as part of the natural evolution of our discipline [11, 30].

Inductive or abductive approaches may be particularly appropriate in at least four situations: (1) when implementation involves emergent or rapidly evolving phenomena (e.g., AI-enabled systems, hybrid care models, digitally mediated work practices) that were not part of the empirical foundations of early TMFs; (2) when studying under-theorized contexts, including non-Western, resource-constrained, or cross-sectoral settings where canonical constructs may not fully capture local dynamics; (3) when existing TMFs fail to adequately explain observed anomalies, tensions, or contradictory findings across studies; and (4) when research seeks to understand temporal dynamics, feedback loops, or mechanism development over time rather than static determinants. In such cases, theory refinement, boundary specification, or mechanism elaboration may be more valuable than deductive verification alone.

Several structural developments in the field further justify renewed emphasis on inductive exploration:

- Empirical maturity in the field has increased, with research now spanning multiple sectors, including education, social care, public health, and digital technology, and diverse cultural contexts [31, 32], creating fertile ground for theory-building that was less prevalent 20 years ago.
- Emergent phenomena and the increase in complex interventions and implementation strategies are not fully accounted for by the core constructs of the TMFs. The increase in hybrid effectiveness–implementation trials, adaptive learning systems, and co-creation methodologies presents situations poorly captured by classic TMFs [9, 19, 33–35].
- Because it examines how people and organizations enact and sustain change, implementation science is fundamentally a social science [36, 37]. As social phenomena resist fixed categorization, the field’s theoretical vitality relies on interpretive sensitivity, the ability to recognize nuance, meaning, and context in human action [23].
- Concerns about epistemic equity arise from the fact that early TMFs originated largely in Western health systems; as empirical contexts expand, there is a growing need for theories that emerge from local practices rather than being taken off the shelf and imported wholesale [38].

Critics may warn that too much inductive proliferation risks fragmentation and loss of cumulative knowledge [39]. If every study develops new concepts, comparison becomes impossible. Moreover, induction without theoretical discipline can devolve into anecdotalism [11].

These concerns are valid but manageable. Inductive inquiry need not abandon theoretical coherence; rather, it can re-ground it. The key is methodological transparency and meta-synthesis. Just as systematic reviews integrate quantitative data, meta-ethnography [40] or realist synthesis [41] can integrate qualitative insights to generate higher-order constructs. A diverse set of emergent theories can then be synthesized into richer mid-range models. Pluralism, therefore, is not the enemy of accumulation but its very precondition.

### Advancing a re-inductive agenda in implementation science

Advancing a re-inductive agenda in implementation science requires aligned changes across the research ecosystem. Choices made at the level of individual studies shape how theory is generated, field-level infrastructures determine whether insights accumulate, and institutional incentives influence what kinds of theory work are valued. The following sections outline how these three levels can work together to support theory development that is adaptive, cumulative, and grounded in practice.

### Study-level strategies

At the level of individual studies, advancing a re-inductive agenda requires loosening the grip of foundational TMFs as fixed taxonomies and reasserting their status as provisional, evolving heuristics. As several scholars have noted, implementation science research has at times treated canonical TMFs with undue reverence, privileging faithful application over empirical responsiveness [13, 16, 42]. Contemporary scholarship instead emphasizes iterative refinement, empirical adaptation, and context-sensitive theorizing, in which theory is reshaped through engagement with practice rather than imposed in advance [11, 14, 43, 44].

Re-embracing qualitative discovery is central to this shift. Ethnographic and grounded approaches enable researchers to observe implementation as it unfolds, capturing how reasoning, interaction, and adaptation emerge in situ [23, 24]. Rather than beginning with predefined constructs such as *relative advantage* or *inner setting*, studies can ask what actually organizes practice in a given context, allowing constructs to emerge inductively from data; these data may then be organized to facilitate comparison with other empirical observations, leveraging extant TMFs where appropriate.

Abductive reasoning provides a methodological bridge between induction and deduction, helping the field avoid both rigid orthodoxy and atheoretical proliferation. By iterating between empirical surprises and theoretical revision, abduction treats anomalies as analytically productive, using unexpected findings to refine or reconfigure theory rather than forcing them into existing categories [29].

Authors can justify inductive or abductive approaches by clearly articulating (a) the theoretical limits of existing TMFs in relation to their empirical setting, including unaddressed constructs, unclear mechanisms, or overly generalized assumptions; (b) specific anomalies, inconsistencies, or tensions identified in prior research that call for explanation rather than confirmation; and (c) the anticipated theoretical contribution, such as refinement of constructs, clarification of boundary conditions, or development of mechanism-based explanations.

Explicitly framing inductive and abductive modes of inquiry as strategies for strengthening and extending theory, rather than abandoning it, may help reviewers recognize its rigor and cumulative intent. At the study level, this implies greater transparency about how TMFs are adapted, extended, or challenged and a reporting culture that values conceptual learning as much as hypothesis confirmation.

Researchers can enhance reviewer receptivity by documenting: (1) which constructs were retained, modified, omitted, or added; (2) what specific empirical observations prompted these adaptations; (3) how adaptations alter construct definitions, relationships, or mechanisms; and (4) whether changes are proposed as context-bound refinements or more general theoretical extensions. Presenting adaptation as cumulative learning rather than deviation may reduce perceptions of theoretical drift and strengthen the clarity of theoretical contribution.

### Field-level strategies

For inductive and abductive insights to accumulate rather than remain isolated, the field requires shared infrastructures that support synthesis and comparison. One promising direction is the development of repositories that catalog emergent constructs generated through inductive and abductive studies. Such repositories could be embedded within journal platforms, professional societies, or collaborative research networks. Contributions might include short, structured concept notes describing emergent constructs, their empirical grounding, contextual scope, and relationship to existing TMFs. These entries could be periodically synthesized through living reviews, theory-focused special issues, or collaborative workshops. Digital concept-mapping platforms could allow researchers to trace how constructs evolve, merge, or differentiate across contexts over time.

Learning communities, formed through conferences, special interest groups, or funded networks, could provide spaces for collective refinement of challenging or ambiguous constructs, enabling dialogue around boundary conditions and mechanism specification. In this way, inductive insights would accumulate rather than remain isolated within single studies. Alongside established TMF domains, such repositories could document newly observed roles (e.g., boundary spanners who translate evidence across professional or organizational boundaries) and relational mechanisms (e.g., sense-making, reciprocal adaptation) that appear repeatedly across settings.

By curating these concepts across contexts, the field could move beyond one-off conceptual innovation toward cumulative mid-range theorizing grounded in real implementation work. Synthesis infrastructures, such as living reviews, concept mapping platforms, or theory-focused meta-ethnographies, would allow researchers to trace how constructs travel, mutate, or stabilize over time and across domains.

A re-inductive field agenda also requires taking temporality seriously. Implementation is rarely linear, yet many studies remain cross-sectional, capturing only a single moment in a dynamic process. Longitudinal programs of work would reveal how implementation unfolds as enthusiasm wanes, strategies are adapted or abandoned, leadership shifts, and new constraints emerge [42]. Tracking these trajectories over months or years would uncover the feedback loops through which organizations learn and reorganize, strengthening theory that accounts for change rather than static conditions.

### Institutional-level strategies

Institutional structures play a decisive role in shaping what kinds of theory work are possible. Journals and funders often call for “theoretically grounded” research, but in practice, this can reinforce a narrow deductive orthodoxy that privileges alignment with established TMFs over conceptual exploration. Paradoxically, the proliferation of TMFs has made editors and reviewers more cautious about new theoretical contributions, further entrenching reliance on canonical frameworks and discouraging innovation.

Advancing a re-inductive agenda, therefore, requires institutional signals that explicitly value theory development, refinement, and transparent adaptation, not just theory application. Editorial policies and review criteria could broaden definitions of rigor to include abductive reasoning, interpretive depth, and reflexivity about theory-building. Funders could support exploratory and longitudinal programs of work that prioritize learning over early closure. By recalibrating incentives in this way, journals and funders can help restore balance between discovery and verification, enabling implementation science to remain theoretically vibrant and avoid unproductive proliferation.

This tension between formal rigor and conceptual contribution is not unique to implementation science. In qualitative methodology, concerns have likewise been raised about overly rigid reporting criteria that risk substituting procedural compliance for conceptual depth (e.g., critiques of checklist-based approaches to qualitative rigor). These debates highlight how formal alignment with reporting standards does not necessarily equate to theoretical contribution or interpretive richness. Evaluating theory-building work therefore requires attention to explanatory insight, reflexivity, and conceptual advancement rather than checklist conformity alone.

### Achieving a balanced logic of inquiry in research practice

Institutional support and favorable field infrastructures can create conditions for change, but the substantive work of rebalancing deduction and induction must occur within everyday research practice. A pressing question, therefore, is how researchers can translate this balanced epistemology into the design and conduct of empirical implementation studies. When substantial a priori knowledge exists, how can data collection and analysis still leave space for inductive discovery?

Implementation science studies benefit from deliberately alternating structure and openness, cycling through complementary reasoning logics across different successive stages of the research process. Initial fieldwork often leans inductively, allowing insights to emerge from practice, yet this openness should be anchored by giving attention to the specific knowledge gaps the study aims to address and the relevant bodies of literature, ensuring the work is informed by previous insights rather than by being purely exploratory. During data analysis, reasoning often becomes abductive because researchers connect empirical discovery with evolving theoretical interpretations. In follow-up studies, a more deductive approach may help test propositions and examine variation across contexts. Across all stages, a calibrated balance between different logics of inquiry is required, and this balance should vary with the project-specific ratio of established knowledge to genuine novelty. This entails allowing previous theoretical knowledge and researcher assumptions to remain in productive dialogue with new empirical insights, which in turn should reshape theoretical understanding to better account for heterogeneous mechanisms, contexts, and real-world implementation dynamics.

This flexibility also demands varying levels of skill and confidence. Early-career implementation science researchers may struggle to judge when to depart from a TMF or how far inductive exploration should proceed before coherence is lost. They would benefit from clearer methodological guidance, mentorship, and exemplars that illustrate this iterative reasoning. In contrast, more experienced researchers in the field are often better placed to take epistemic risks, emphasizing reflexivity, documenting adaptation processes, and supporting others in developing these capabilities.

Across the implementation science field, researchers could make their studies more valuable by reporting clearly when and how they adapt TMFs and what specific empirical findings or challenges prompted those adjustments. For example, noting when a construct needs reinterpretation or when new contextual factors demand additions can help transform the use of theory into its ongoing refinement. We can also learn from other social science disciplines that place an emphasis on making a theoretical contribution from empirical studies, with a long history of reflexive observations on what such contributions entail and how they can be fostered [28, 45]. Over time, such cumulative reflexivity would create a richer, context-sensitive evidence base, showing that theoretical coherence and empirical openness are not opposing goals but mutually reinforcing ones. These shifts call not for the abandonment of established TMFs, but for their continuous, transparent renewal in practice.

Less experienced researchers may benefit from explicitly planning staged reasoning strategies, for example, beginning with open-ended data collection before mapping findings to TMFs; maintaining analytic memos that document tensions between data and theory; engaging in peer debriefing or mentorship when considering theoretical departures; and pre-specifying in study protocols how inductive insights will be evaluated in relation to existing frameworks. Publishing reflexive methodological appendices that document these processes may provide reassurance to reviewers that departures from canonical TMFs are theoretically grounded rather than arbitrary.

### What inductive renewal looks like in practice: illustrative examples

The argument advanced in this paper is not that established TMFs should be abandoned, but that their repeated deductive application risks constraining theoretical development if not complemented by inductive and abductive forms of inquiry. A natural question, therefore, is what such a renewal looks like in practice and how it differs from business-as-usual applications of TMFs. The following illustrative examples contrast conventional deductive uses of TMFs with alternative approaches that put empirical discovery and reflexive theory development in the foreground, while preserving conceptual coherence.

Research by Kislov and colleagues on knowledge brokering as an implementation strategy illustrates how inductive discovery can be combined productively with deductive anchoring through a distinctly abductive “back-and-forth” between empirical puzzles and conceptual refinement. An inductive multiple-case study [46] revealed the strategies through which professionals with managerial responsibilities enacted brokering in situ, and abductive reasoning was used to reflect on these emergent strategies in relation to Ward et al.’s [47] typology of knowledge brokering and the literature on professional hybrids.

In another study by Kislov and colleagues [48] abduction reasoning is even more apparent; the empirical puzzle of how knowledge brokers’ legitimacy shifts over time prompted the selection of Bourdieu’s theory of practice [49] as an explanatory lens, not as a coding framework to be applied, but as a means of developing mechanism-based explanations of evolving struggles over legitimacy, including the trade-offs and compromises involved.

In the analysis of facilitation as an “enabling” dimension of knowledge brokering [50], three mechanisms were theorized regarding how facilitation can become “distorted” as implementation unfolds across stages. Although driven by longitudinal observations of declining implementation fidelity, this analysis was theoretically elaborated through engagement with literature on the evolution of managerial techniques and on facilitation as a theory of learning.

Redwood et al. [51] described a more recent example of theoretically informative work in this journal involving a qualitative process evaluation of the national PReCePT (PReventing Cerebral Palsy in pre-Term labour) program scale-up, which makes its reasoning strategy explicit. Inductive sense-making was used to stay close to what implementers actually did, and abductive iteration interrogated these observations through a deductive lens provided by NPT [6]. The study began with an inductive framework analysis, enabling systematic comparison across maternity units and generating a structured account of how implementation resources were mobilized in practice. Rather than stopping at a descriptive catalog of contextual factors, the authors then deployed abduction to develop explanations by engaging empirically with NPT, focusing on how the strategic aim of increasing magnesium sulfate administration was translated into new forms of everyday work organization.

This abductive engagement sharpened the study’s theoretical contribution: NPT constructs were mobilized to distinguish implementation outcomes, namely *normative restructuring*, *relational restructuring*, and *sustainment*, and to explain why similar uptake gains could conceal important differences in how changes were stabilized once additional resources were withdrawn. The result is not merely an “application” of NPT as an established TMF, but a more discriminating, mechanism-attentive account of scale-up grounded in sustained empirical engagement with a subset of NPT constructs.

These examples illustrate what a balanced inductive–abductive–deductive logic of inquiry looks like in practice. Across these studies, theoretical advancement arises not from the repeated deductive application of established TMFs, but from their reflexive refinement through sustained empirical engagement. Previous theory is neither treated as a fixed template nor abandoned altogether; instead, it is selectively mobilized to structure comparison, sharpen explanation, and support conceptual development. Abductive iteration plays a central role in translating empirical surprises, temporal shifts, and analytic tensions into mechanism-attentive and generalizable accounts of how knowledge brokering and implementation scale-up operate as collectively enacted phenomena. In doing so, these contributions preserve theoretical coherence while enabling genuine conceptual renewal.

### Preserving TMFs when renewing them: a call for reflexive theory development

A call for greater inductive exploration is not a rejection of established TMFs. Foundational approaches such as CFIR [5] and TDF [7] remain highly valuable, but they are best understood as heuristics rather than doctrine. Recent updates to major TMFs, such as the revised CFIR [52], explicitly acknowledge the need for openness to emergent constructs and contextual adaptation. We view these developments as important and constructive steps. However, encouragement alone may be insufficient unless accompanied by broader methodological, cultural, and institutional shifts that normalize theory refinement and adaptation as core scholarly contributions rather than discretionary departures from orthodoxy.

The usefulness of TMFs can be preserved and renewed through several strategies:

- Comparative theoretical analysis: systematically contrast how different TMFs interpret the same data to expose conceptual blind spots.
- Context-sensitive adaptation: treat TMFs as context-dependent and, where needed, adapt them by specifying boundary conditions under which their assumptions hold and/or refine constructs, relationships, and mechanisms to reflect contextual contingencies observed in the data.
- Integration through metatheory: use higher-level sociological or systems theories (e.g., structuration, complexity, institutional work) to situate specific TMFs within broader explanatory logics.
- Cross-pollination of theory: draw on useful theoretical strands from sociology, organization studies, and related fields, recognizing that novelty often emerges when ideas travel across disciplines or take shape in the spaces between them.

Such strategies treat theory as both cumulative and revisionary. TMFs should be continually tested, extended, cross-fertilized, and re-contextualized rather than simply re-used.

## Conclusion

Implementation science will generate useful insights not only by applying theory but by advancing it. Early TMFs provided coherence at a formative stage in the field, but their uncritical, repeated application now risks intellectual stagnation. A mature discipline must periodically return to empirical phenomena to rediscover, re-interpret, and re-theorize them [53].

This requires a balanced logic of inquiry with deduction grounded in robust TMFs, continually challenged, and refreshed through induction and abduction. Accumulation of practically relevant, generalizable knowledge is possible only if the field remains self-reflexively inductive, sensitive to contexts, anomalies, emergent realities, and lived experiences that resist neat theoretical categorization.

In short, implementation science should move beyond asking “Which TMF fits my project?” and instead ask “What does this phenomenon reveal that existing TMFs cannot yet explain?” and “What refinements to our core TMFs does this phenomenon suggest?” Framing inquiry in this way helps ensure that the field remains both empirically grounded and theoretically generative.

## Data Availability

No datasets were generated or analysed during the current study.
